# Discussion on advancing the methods for quality improvement research

**DOI:** 10.1186/1748-5908-8-S1-S10

**Published:** 2013-04-19

**Authors:** Patricia S Groves, Theodore Speroff, Paul V Miles, Mark E Splaine, Denise Dougherty, Brian S Mittman

**Affiliations:** 1 Veterans Affairs National Quality Scholars Program, Iowa City VA Medical Center, Iowa City, Iowa, 52246, USA; 2 University of Iowa College of Nursing, Iowa City, Iowa, 52242, USA; 3 Center for Health Services Research, Geriatric Research, Education, and Clinical Center (GRECC), Veterans Affairs Tennessee Valley Healthcare System, Nashville, Tennessee, 37212, USA; 4 Vanderbilt University School of Medicine, Nashville, Tennessee, 37232, USA; 5 American Board of Pediatrics, Chapel Hill, North Carolina, 27514, USA; 6 Geisel School of Medicine at Dartmouth, Hanover, New Hampshire, 03755, USA; 7 Agency for Healthcare Research and Quality, Rockville, Maryland, 20850, USA; 8 VA Center for the Study of Healthcare Provider Behavior, Veterans Affairs Greater Los Angeles Healthcare System, Sepulveda, California, 91343, USA

## Introduction

Our conference on advancing the methods of healthcare quality improvement research provided an opportunity to reflect on the progress of the quality improvement movement over the past two decades and to assess the current status of this field. In this paper, we highlight some meaningful themes that emerged across presenters and echo those voiced by attendees during roundtable discussions.

## Current status

### Quality improvement research is an increasingly productive field of study

The development of study design and methods illustrated by meeting speakers, abstracts, and posters indicates that quality improvement research is coalescing into a discipline of study. Research now appears in a broad range of impact journals (e.g., New England Journal of Medicine, Annals of Internal Medicine, Pediatrics), and journals specific to patient safety, implementation science, and quality improvement are now mainstream. There are now strong research designs and analytic methods available for quality improvement researchers. Studies presented during the Conference demonstrate the emerging use of cluster randomized controlled trials, hybrid clinical effectiveness trials, planned-experiments using fractional factorial design, complex regression, and realistic evaluation analysis. Finally, Standards for Quality Improvement Reporting Excellence (SQUIRE) publication guidelines provide criteria for formulating and disseminating this research.

### The science of quality improvement has reached a tipping point

Health professions currently have advanced fellowships for career pathways in pragmatic research and quality improvement. There has been a groundswell of support promoted through private, public, professional, and government agencies for a quality scholar workforce. Competency in quality improvement is now required for all physicians to maintain board certification, and quality and safety are required components of baccalaureate and doctoral nursing education. Indeed, the field of quality improvement embraces variation, context, and culture; an understanding of these are certainly needed for a healthcare system on the brink of change [1,2]. Quality improvement research is poised to address questions pertinent to healthcare reform and better delivery of care through methods appropriate for the study of complex, multi-level, system-wide interventions.

Registries for healthcare quality are rapidly emerging as tools for practice-based learning networks and multi-site improvement learning collaboratives. Statisticians have developed powerful approaches to draw robust conclusions in the presence of confounders, variable case mix, and varying risk factors. New practice-based networks have packaged best practices into bundles for replication and spread of quality improvement. Methods workshops and initiatives for innovation have become features at professional organizations (e.g., Academy for Healthcare Improvement, Academy Health, Society for Hospital Medicine, Veterans Affairs QUERI), funding agencies (e.g., Agency for Healthcare Research and Quality [AHRQ], Robert Wood Johnson Foundation), and government programs (e.g., Centers for Medicare and Medicaid Services). Healthcare organizations are learning to use these tools and data while transforming to accountable, learning organizations.

In sum, quality improvement research is gaining momentum. The tools and instruments used in quality improvement are maturing and the science is contributing innovative methods, such as realistic evaluation, complex simulation modeling, and closed-loop dynamic systems engineering. There is a critical mass of scholarship in the form of training for healthcare professionals, quality improvement curricula, and fellowships. Concurrently, environmental pressure for healthcare reform is priming a culture change.

## Challenges

### Need for unifying theory and language

Although we have a vast amount of published quality improvement literature and have revamped health professions curricula, the field is fragmented and scattered. At times, quality improvement seems more like a flavor of the month than a conceptual framework with foundational concepts and causal pathways that can be used to produce generalizable knowledge. Quality improvement research encompasses fields of healthcare, management, social science, and education. The socialization and jargon of scientists from different disciplines results in confusing terminology, difficulties in definition, and multiple dialects expressing various models of quality improvement. Furthermore, quality improvement is often viewed as a parochial tool for solving discrete problems, and not for pursuing scientific, generalizable knowledge. Such misunderstandings are a threat to the acceptance of a quality improvement research discipline.

### Understanding and relevance

Discourse defining quality improvement is happening primarily among academics in this nascent field. The lack of a unified conceptual framework and vocabulary limits the lay community’s comprehension – in fact, the term “quality improvement research” does not even appear in Wikipedia. There seems to be relatively little understanding of what works to improve quality and how and why interventions may vary in effectiveness across settings. In order for quality improvement research to be seen as part of the legitimate solution to the problems facing healthcare, it is essential and incumbent upon the professionals in the field to provide an understanding to patients, the community, industry, funders, and government about the nature and relevance of quality improvement research.

## Future opportunities

In addition to National Institutes of Health and AHRQ funding, new opportunities for quality improvement researchers to demonstrate relevance to patients, families, stakeholders, and policy makers include the high profile Patient Centered Outcomes Research Initiative [http://www.pcori.org] and Medicare innovation projects [http://www.innovations.cms.gov]. Quality improvement researchers should engage in these endeavors with the goals of proving the worth of their field and gaining the understanding of patients and policy makers of their work’s value. The Conference illuminated the potential role of quality improvement research in several ways, including thorough presentations on comparative effectiveness, disparities, and government contracting. In addition, it is time to push healthcare delivery systems towards quality improvement research by aligning research departments with frontline clinicians to (a) systematically analyze quality issues, (b) implement innovative quality improvement tools, and (c) evaluate their effectiveness in different contexts.

In conclusion, quality improvement researchers need to actively shape the field. A conceptual framework and vocabulary are needed to define the field and its paradigm. An international federation of quality improvement researchers and experts working collaboratively with implementation science and other associated fields should mobilize to advocate the distinctive methodologies for improving quality of care. Endeavors to formulate theory and testable hypotheses, conduct studies that generate meaningful generalizable knowledge, create and test process and outcome quality measures, and bring about return on investment, are all on the horizon for advancing quality improvement research.
